# What Impact Do Medicolegal Complaints Have on Canadian Plastic Surgeons?

**DOI:** 10.1177/22925503241300337

**Published:** 2024-12-05

**Authors:** Zach Zhang, Hassan El Hawary, Paul Oxley, Mirko S. Gilardino, Jugpal S. Arneja

**Affiliations:** 1 Division of Plastic and Reconstructive Surgery, Department of Surgery, 8166 University of British Columbia, Vancouver, Canada; 2 Division of Plastic and Reconstructive Surgery, Department of Surgery, 5620 McGill University, Montreal, Canada; 3 Division of Plastic and Reconstructive Surgery, Surrey Memorial Hospital, 8166 University of British Columbia, Vancouver, Canada

**Keywords:** medicolegal, plastic surgery, malpractice, Médico-légal, chirurgie plastique, faute professionnelle

## Abstract

**Background:** Being named in a medicolegal complaint can be a lengthy process filled with uncertainties. Its current impact on Canadian plastic surgeons is unknown. We aim to review the impact of medicolegal complaints and provide advice for surgeons on how to prevent, minimize, and navigate through the medicolegal process. **Method:** An anonymous survey was sent to registered Royal College of Physicians and Surgeons of Canada certified plastic surgeon members. The survey collected data on surgeon demographics (clinical status, gender, practice type, volume), medicolegal complaint history and outcome, impact on practice, and insights into the process. Surgeons with an inactive practice and invalid contact information were excluded. **Results:** Out of 456 invited plastic surgeons, 100 responses were included, 73% were male, with an even distribution of practice types and years in practice. Most were Canadian Medical Protective Association (CMPA) members. A significant portion (62%) had been named in a medicolegal action, primarily related to treatment complications (42%) and poor outcomes/disease progression (34%). Factors associated with complaints were greater years in practice (*P*<.01), and a higher annual volume of operating room cases (*P*=.02). The duration of the medicolegal process varied, with the majority taking 1-2 years. Respondents predominantly agreed that CMPA provided adequate legal defence (83%, 53/64). However, most surgeons felt the process had a significantly negative impact on their mental health (75%, 48/64). After being involved in a complaint, many surgeons modified their practice pattern by increasing documentation/consent process (45%, 29/64), avoiding certain procedures (22%, 14/64), and avoiding care of high-risk patients (19%, 12/64). **Conclusion:** Despite legal resolution in favour of the physicians, the results of this survey indicate the medicolegal complaint process has a significant impact on plastic surgeons’ practices, time, and mental health. Understanding the medicolegal process and outcome is crucial for risk mitigation.

## Résumé

**Contexte :** Être cité dans une plainte médico-légale peut être un long processus rempli d’incertitudes. Son impact actuel sur les chirurgiens plasticiens canadiens est inconnu. Nous avons cherché à étudier l’impact des plaintes médico-légales et à fournir des conseils aux chirurgiens sur la manière de prévenir, minimiser et naviguer dans le processus médico-légal. **Méthode :** Une enquête anonyme a été envoyée aux membres du Collège royal des médecins et chirurgiens du Canada certifiés en chirurgie plastique. L’enquête a collecté des données sur les données démographiques du chirurgien (statut clinique, genre, type de pratique, volume), les antécédents de plainte médico-légale et leur aboutissement, l’impact sur la pratique et les points de vue sur la procédure. Les chirurgiens n’exerçant pas ou ayant des coordonnées non valides ont été exclus de l’enquête. **Résultats :** Sur les 456 chirurgiens plasticiens invités, 100 réponses ont été incluses : 73% des répondants étaient des hommes, avec des types de pratique et un nombre d’années d’activité également répartis. La majorité était membres de l’ACPM (Association canadienne de protection médicale). Un pourcentage significatif (62%) des répondants a été cité dans une action médico-légale, principalement liée à des complications du traitement (42%) et à des résultats médiocres/une progression de la maladie (34%). Les facteurs associés aux plaintes étaient un plus grand nombre d’années d’exercice (*P* < 0,01) et un plus grand nombre annuel de cas opératoires (*P* = 0,01). La durée de la procédure médico-légale était variable, la majorité a duré de 1 à 2 ans. Les répondants ont reconnu pour leur plus grand nombre que l’ACPM avait fourni une défense juridique appropriée (83%, 53/64). Toutefois, la plupart des chirurgiens ont estimé que le processus avait eu une répercussion significativement négative sur leur santé mentale (75%, 48/64). Après avoir été impliqués dans une plainte, de nombreux chirurgiens ont modifié leurs méthodes de travail en augmentant le processus de documentation/consentement (45%, 29/64), en évitant certaines procédures (22%, 14/64) et en évitant de traiter des patients à risque élevé (19%, 12/64). **Conclusion :** En dépit d’une résolution juridique favorable aux médecins, les résultats de cette enquête indiquent que le processus de plainte médico-légale a une répercussion significative sur la pratique, le temps et la santé mentale des chirurgiens plasticiens. La compréhension du processus médico-légal et de son aboutissement est essentielle pour une limitation des risques.

## Background

Medical malpractice constitutes a significant cost of healthcare, with spending reaching 2.4% of total health care expenditure in the United States.^1^ The Canadian Medical Protective Association (CMPA) reports an 85% increase over the past decade in requests for assistance.^2,3^ Recently published CMPA data shows the annual incidence of College complaint among practicing plastics surgeons is 12% and civil-legal action is 7%.^4^ Medicolegal complaints have become a relatively common occurrence in the career of a plastic surgeon. However, there is a paucity of literature that explores the impact of such complaints on Canadian plastic surgeons’ practices. This study aims to fill this gap by obtaining a subjective evaluation of medicolegal complaints from the perspective of Canadian plastic surgeons.

## Methods

Research ethics approval was obtained from University of British Columbia Children's and Women's Research Ethics Board (identification number: H22-00866). An anonymous survey was designed to assess the pattern and impact of medicolegal complaints among Canadian plastic surgeons. All registered Royal College of Physicians and Surgeons of Canada certified plastic surgeon members of the Canadian Society of Plastic Surgeons (CSPS) and Canadian Society for Aesthetic Plastic Surgery (CSAPS) were sent an invitation with three reminders over the period of a month, one week apart, to complete an online questionnaire through the UBC survey platform—Qualtrics. The invitation included a cover letter explaining the purpose of the study. The survey collected data on surgeon demographics (years in practice, gender, practice type, volume), medicolegal complaint history (frequency, type, reason, practice change), impact on practice and insights into the process. We excluded any surgeon with an inactive practice, invalid contact information, and incomplete responses.

### Statistical Analysis

Data analyses were conducted with IBM SPSS Statistics Version 27 software. Descriptive analyses were performed for all variables and reported as percentages. Chi-square tests were performed as univariate analysis to identify factors significantly associated with medicolegal complaints. Significant variables were then included in the multivariate analysis using a covariance model. Open-ended answers to questions were categorized to identify the most common themes.

## Results

### Respondent Demographics

Out of 456 unique CSPS and CSAPS members invited, 108 surgeons responded, with 8 exclusions due to incomplete responses. The overall response rate was 22% (100/456). Of the 100 included respondents, 73% were male. Common practice types were well-represented including academic (39%), private (33%), and community-based (28%). The sample represented surgeons from 8/10 provinces (Alberta, British Columbia, Manitoba, New Brunswick, Nova Scotia, Ontario, Quebec, and Saskatchewan) and 1/3 territories (Nunavut). There was an even distribution of years in practice among the respondents—<10 years (20%), 11-20 years (28%), 21-30 years (23%) to >30 years (29%). The vast majority of the respondents reported having a CMPA membership (99%). Thirteen surgeons (13%) reported having additional private malpractice insurance outside of CMPA (Table 1).

**Table 1.** Respondent Demographics, Clinical Status, Practice Pattern, and History of Medicolegal Complaints.

Variable	n (%)
Gender	
Male	73 (73%)
Female	23 (23%)
Clinical status (years in practice)	
< 5 years	7 (7%)
5-10 years	13 (13%)
11-20 years	28 (28%)
21-30 years	23 (23%)
> 30 years	29 (29%)
Predominant type of practice	
Academic	39 (39%)
Community	26 (26%)
Private practice	33 (33%)
Prefer not to disclose	1 (1%)
Proportion of aesthetic practice (by time)	
None	17 (17%)
1%-25%	34 (34%)
26%-50%	14 (14%)
51%-75%	12 (12%)
> 75%	23 (23%)
Practice location	
British Columbia	26 (26%)
Alberta	15 (15%)
Ontario	28 (28%)
Quebec	19 (19%)
Other provinces/territories	12 (12%)
CMPA membership	
Yes	98 (98%)
No	1 (1%)
Prefer not to disclose	1 (1%)
Malpractice insurance outside of CMPA	
Yes ($1-$9999/year)	6 (6%)
Yes ($10,000-$19,999/year)	0 (%)
Yes ($20,000-$29,999/year)	3 (3%)
Yes (≥ $30,000/year)	3 (3%)
No	86 (86%)
Prefer not to disclose	1 (1%)
Medicolegal action	
Yes	62 (62%)
No	38 (38%)

### Medico-Legal Complaint Pattern

The majority of the respondents (n=62/100; 62%) were named in a medicolegal action. Out of these respondents, 39% were named once, 21% twice, 27% three times, and 13% four or more times. The most common reasons for complaints were related to treatment complications (42%) and poor outcome/disease progression (34%).

### Impact on Surgeons

The duration of the medico-legal process was found to have a wide range—<1 year (16%), 1-2 years (45%), 3-5 years (26%), >5 years (13%). The majority of surgeons spent between 1-10 h (42%), and 11-25 h (40%) on their defense. Most respondents paid no direct legal fees (98%), but one respondent needed to spend between $10,000 and 25,000 CAD.

Most respondents felt the outcome of the medicolegal complaint was fair (75%). The majority agreed that the CMPA provided adequate legal defense (83%). However, most surgeons felt the process had a significantly negative impact on their mental health (77%). Many also felt that being involved in a medicolegal case caused significant loss of time (61%). Few respondents felt that the process had an impact on financial income (10%), career trajectory (5%), and personal relationships (15%). After being involved in a complaint, some surgeons modified their practice patterns by increasing documentation/consent process (47%), avoiding certain procedures (23%), and avoiding care of high-risk patients (19%).

### Factors Associated With Medico-Legal Complaints

Univariate analysis showed male surgeon gender (*P*=.03), greater years in practice (*P*<.01), greater annual volume of operating room cases (*P*=.02) were associated with medicolegal complaints. Multivariate analysis revealed that only greater years in practice (*P*<.01) and greater annual volume of operating room cases (*P*<.01) remained significant. Variables not significantly associated with medicolegal complaints were annual volume of minor surgery cases (*P*=.33), annual volume of non-operative procedures/injection (*P*=.81), academic versus community practice (*P*=.47), volume of aesthetic practice (*P*=.30), practice province (*P*=.51), and having malpractice insurance outside of CMPA (*P*=.16).

### Comments and Advice

Comments from surgeons focused on the emotional and mental stress, time loss, uncertainty, and length of the process. A total of 39 comments were related to stress and 16 were related to time. Many feared judgment and being viewed as guilty and incompetent until proof of innocence. Others voiced their frustration at the accusations which they perceived to be unwarranted, while some felt significant impacts on their self-confidence and their surgical skills.

Advice was centered around documentation with focus around consent and complications, and understanding the process with the CMPA. Overall, surgeons found the most helpful methods to decrease their stress was talking to colleagues and friends (43%), and speaking to their lawyers (27%).

## Discussion

Despite recent literature on the statistics of Canadian plastic surgery medicolegal complaints,^4,5^ there is a gap in understanding their impact on surgeons. This study highlights the commonality of such complaints in a plastic surgeon's career, suggesting a correlation with volume and years in practice. Although respondents felt they received protection they deemed adequate, the associated stress is challenging. Understanding the medicolegal process can help surgeons mitigate risks and reduce future complaints.

### Medicolegal Process and Outcome

The survey results showed a wide-ranging duration of the medicolegal process. Previous data showed that less than 2 out of 50 annual cases go to trial.^4–6^ Most of our respondents were satisfied with the legal protection provided by the CMPA and the legal outcome. However, previous court data showed that of the small number of cases that do reach trial, surgeon outcome worsens with only one-third of cases ruling in their favour.^5^

### Mental Health Impact

Respondents revealed the process had a significant impact on their mental health. The feeling of loss of control and assault on personal honor is well described as medical malpractice stress syndrome.^7,8^ Symptoms of this syndrome are similar to ones experienced in post-trauma stress disorder. It is important for surgeons to recognize that being involved in a medicolegal complaint does not necessarily mean one is at fault. Ultimately, among our respondents, most medicolegal complaints did not significantly impact career trajectories, financial livelihoods, and personal relationships. The stress and guilt felt during a medicolegal complaint may be reduced by understanding the legal process, building a strong rapport with one's lawyer, and reaching out to peers.^9^ However, it is important to speak with the CMPA prior to speaking with colleagues as any disclosure outside of legal counsel may be detrimental to a potential legal proceeding. More information on the medicolegal process for plastic surgeons is available from papers published by El Hawary *et al* and our group.^4,5^

### Practice Change

The survey results indicated that being involved in a medicolegal complaint prompted changes in practice patterns for nearly half of the respondents. Increased patient education and documentation and focus on complications were common adaptations. This finding is supported by previous studies showing lack of informed consent to be the most common reason for medicolegal complaints.^4,5,10–12^

### Strength and Limitations

This study, the first of its kind for Canadian plastic surgeons, provides qualitative insights in addition to existing data. However, limitations include survey design, self-respondent bias, and potential recall bias. It is unclear if prior medicolegal complaint history would increase or decrease response rate. Generalizability to other surgical specialties and countries is also uncertain.

## Conclusion

Despite protection that plastic surgeons deem adequate by the CPMA and a small proportion of cases going onto trial, medicolegal complaints have a significant impact on plastic surgeons’ practices, time, and mental health. A deeper understanding of the medicolegal process can empower surgeons to navigate these challenges, reduce complaints, and enhance patient satisfaction and safety.
